# Efficacy of neoadjuvant hormonal therapy combined with robot-assisted radical prostatectomy for oligometastatic prostate cancer: a multicenter retrospective study

**DOI:** 10.3389/fonc.2026.1765517

**Published:** 2026-03-26

**Authors:** Yujie Dong, Zhuoran Li, Zhiqiang Chen, Zhen Tian, Weilin Chen, Jieping Hu, Yuqi Jia, Jin Luo, Nanxin Zou, Jinqiao Li, Qiwei Liu, Qiming Yang, Chao Wang, Xiaofeng Xu, Bin Fu, Xu Zhang, Baojun Wang

**Affiliations:** 1 Senior Department of Urology, Chinese PLA General Hospital, Beijing, China; 2 Department of Urology, The First Affiliated Hospital, Soochow Medical College, Soochow University, Soochow, Jiangsu, China; 3 Department of Urology, The First Affiliated Hospital, Jiangxi Medical College, Nanchang University, Nanchang, Jiangxi, China

**Keywords:** neoadjuvant hormonal therapy, oligometastatic prostate cancer, oncological outcomes, perioperative outcomes, robot-assisted radical prostatectomy

## Abstract

**Objective:**

To evaluate the efficacy of neoadjuvant hormonal therapy (NHT) on perioperative and oncological outcomes in patients with oligometastatic prostate cancer (OmPCa) treated with robot-assisted radical prostatectomy (RARP) and adjuvant androgen-deprivation therapy (ADT).

**Methods:**

In this multicenter retrospective study, 160 OmPCa patients treated from five Chinese medical centers between May 2010 and May 2023 were included: 80 received NHT followed by RARP+ADT, and 80 underwent RARP+ADT alone. We evaluated surgical information, pathological findings, biochemical progression-free survival (bPFS), radiological progression-free survival (rPFS), overall survival (OS), subsequent treatments, and ADT-related adverse events.

**Results:**

Compared to the standard therapy (ST) group, the NHT group exhibited shorter operative time (170 vs. 200min; P = 0.030), less blood loss (100 vs. 200 mL; P = 0.022), lower positive surgical margin rate (17.50% vs. 37.50%; P = 0.005), and higher rates of pathological downstaging in T-stage (48.8% vs. 6.3%; P<0.001). More NHT patients achieved undetectable PSA (83.8% vs. 65.0%; P = 0.007) with a shorter time to PSA nadir (2 vs. 3 months; P = 0.002). After a median postoperative follow-up of 52 months, no significant differences were observed in bPFS, rPFS or OS between groups. However, subgroup analysis revealed significant interaction effects between NHT and biopsy Gleason score ≥8, seminal vesicle invasion, and clinical T stage ≥ T3 (all P-interaction<0.05), with these subgroups showing significantly improved bPFS, rPFS and OS. Furthermore, patients in the NHT group received fewer subsequent treatments (32.5% vs. 47.5%, P = 0.031), and the incidence of ADT-related adverse events was comparable between the two groups (all P >0.05).

**Conclusion:**

NHT improves perioperative outcomes and reduces the need for subsequent salvage therapies in OmPCa patients undergoing RARP. It may offer oncological benefits, particularly those with a biopsy Gleason score ≥8 or clinical T-stage≥T3. It represents a safe and feasible neoadjuvant approach.

## 1 Introduction

The concept of “oligometastasis” was first proposed by Hellman and Weichselbaum in 1995 (1). This theory posits that oligometastasis represents an intermediate state between localized disease and widespread metastasis (2). Although distant metastasis has occurred, it remains potentially curable (3), thus warranting aggressive treatment (4–6). Based on this paradigm, radical prostatectomy (RP) combined with androgen-deprivation therapy (ADT) has recently been employed for patients with oligometastatic prostate cancer (OmPCa) to improve survival outcomes (7–10). However, whether further reduction of tumor burden could optimize patient survival has become a key clinical focus.

A previous study has confirmed that neoadjuvant chemohormonal therapy can significantly improve biochemical progression-free survival (bPFS) and radiographic progression-free survival (rPFS) in patients with OmPCa (11). However, the use of chemotherapeutic agents such as docetaxel may lead to myelosuppression-related adverse events including neutropenia. Neoadjuvant hormonal therapy (NHT) can reduce the volume of the primary tumor, lower the pathological stage, and decrease the positive surgical margin rate in prostate cancer (PCa), thereby providing surgical opportunities for patients initially diagnosed with unresectable tumors. Nevertheless, the efficacy of NHT on oncological outcomes in PCa patients remains controversial (12–15). Therefore, whether NHT alone can provide survival benefits for OmPCa patients requires further investigation.

To address this knowledge gap, this study—representing the largest multicenter retrospective clinical investigation globally to date—aims to analyze the efficacy of NHT in OmPCa patients undergoing robot-assisted radical prostatectomy (RARP) with postoperative ADT.

## 2 Materials and methods

### 2.1 Study cohort

This multicenter retrospective study enrolled 160 OmPCa patients treated between May 2010 and May 2023 at the urology departments of five tertiary hospital centers in China (The First, Third, Sixth Medical Centers of Chinese PLA General Hospital, the First Affiliated Hospital of Nanchang University and the First Affiliated Hospital of Soochow University). Among them, 80 patients received NHT combined with RARP followed by postoperative ADT (NHT group), while 80 patients underwent RARP alone plus postoperative ADT (standard therapy [ST] group).

The study complied with applicable laws, Good Clinical Practice, and ethical principles of the Declaration of Helsinki. The protocol was approved by the Ethics Committee of Chinese PLA General Hospital (S2025-406-01). All eligible patients provided written informed consent.

Inclusion criteria: (1) Pathologically confirmed PCa post-surgery; (2) Imaging confirmation (via magnetic resonance imaging [MRI] combined with positron emission tomography/computed tomography [PET/CT]) of ≤5 bone metastases defining OmPCa; (3) Absence of major surgical contraindications (e.g., anesthesia intolerance or severe coagulopathy); (4) Primary diagnosed cases. Exclusion criteria: (1) >5 bone metastases or visceral metastases on MRI combined with PET-CT; (2) Preoperative treatments other than ADT; (3) Open or laparoscopic RP performed after diagnosis; (4) Concurrent malignancies; (5) History of transurethral prostatectomy; (6) Incomplete clinical data.

Given the retrospective and multicenter design of this study, the types of PET tracers used varied across centers and over the study period. A detailed breakdown of PET tracer types by center and time period is provided in **Supplementary Table 1**.

Patients in the NHT group received ADT prior to surgery. The treatment regimen consisted of a subcutaneous injection of 3.6 mg goserelin every 28 days, or an intramuscular injection of 3.75 mg leuprorelin or 3.75 mg triptorelin every months, with or without daily oral bicalutamide 50 mg. The decision to combine bicalutamide was determined by urologists based on a comprehensive evaluation of individual patient factors in accordance with EAU guidelines (16). Serum prostate-specific antigen (PSA) levels were monitored monthly during the treatment period. Both groups of patients underwent RARP along with postoperative lifelong ADT. All surgical procedures were performed by experienced senior surgeons. Subsequent treatment plans were formulated based on the patient’s performance status, comorbidities, and personal preferences, and were ultimately determined by urologists following multidisciplinary team (MDT) discussions.

### 2.2 Outcome measures

(1) Perioperative outcomes: Operative time (skin incision to final suture), intraoperative blood loss, transfusion volume, postoperative hospital stay (discharge criteria: tolerating liquid diet and drain removal), drainage tube duration (removal criteria: effluent <50 mL/day and clear), catheterization duration, perioperative complications (recorded by Clavien-Dindo classification), and positive surgical margin (PSM) status. (2) Oncological outcomes: PSA nadir achievement, bPFS, rPFS, and overall survival (OS). (3) ADT-related adverse events were documented using the National Cancer Institute Common Terminology Criteria for Adverse Events (CTCAE), version 5.0. To maximize consistency, two independent researchers classified the events according to the CTCAE v5.0 criteria. Any discrepancies were resolved through adjudication by a third senior physician.

PSM was defined as malignant glands at inked resection margins. Pathological complete response (pCR) was defined as the absence of any viable tumor cells within the prostate specimen. Minimal residual disease (MRD) was defined as the presence of residual tumor with a maximum dimension of less than 5 mm. Pathological downstaging of T stage was defined as a lower pathological T stage postoperatively compared to the clinical T stage assessed preoperatively via MRI combined with PET/CT. Perioperative complications occurred within 30 days postoperatively. The radical-level PSA was defined as “undetectable” PSA after initial treatment. The nadir PSA was defined as the lowest level that PSA drops after initial treatment. bPFS was calculated from the date of surgery to biochemical recurrence (BCR) or death. BCR is defined as: a) PSA ≥0.2 ng/mL confirmed twice ≥2 weeks apart after achieving radical-level PSA, or b) ≥50% increase from nadir in two consecutive measurements ≥1 week apart if PSA remained detectable. rPFS spanned from the date of surgery to radiographic progression or death. Radiographic progression was defined as the appearance of ≥2 new bone lesions or 1 new soft tissue lesion (bone metastatic lesions are evaluated according to the PCWG-2 criteria, and soft tissue lesions are evaluated according to the RECIST 1.1 criteria (17)). OS was defined as the time from the date of surgery to death from any cause.

The identification and location of metastatic lesions were initially determined by local investigators at each participating center. To ensure consistency, imaging studies from all enrolled patients were subsequently subjected to a centralized, retrospective review by two radiologists who were blinded to the clinical outcomes. In cases of discrepancy between the local report and the central review, a consensus was reached through discussion. During postoperative follow-up, MRI was performed at prespecified intervals: every three months for the first two years, every six months from year three to year five, and annually thereafter. Additional scans were conducted as clinically indicated, such as in the event of a PSA rise or new-onset symptoms. For suspicious new lesions, confirmation by PET/CT was required within four weeks; rPFS was defined only when at least two imaging modalities indicated progression. Follow-up for other oncological outcomes was performed every three months during the first two postoperative years, every six months from year three to year five, and annually thereafter. The follow-up ended in June 2025.

### 2.3 Statistical analysis

Continuous data were reported as mean ± standard deviation or median (Q1, Q3); categorical data as counts (percentages). Intragroup comparisons used paired t-tests. Intergroup comparisons: independent t-tests for normally distributed data, Wilcoxon rank-sum tests otherwise; χ² or Fisher’s exact tests for categorical variables. Kaplan-Meier curves depicted time-to-event outcomes, compared by log-rank tests with hazard ratios (HR) and 95% confidence intervals (CI). Univariate and multivariate Cox proportional hazards regression models were used to analyze predictors of bPFS, rPFS, and OS. Variables with a P-value < 0.1 in univariate analysis were included in the multivariate model to identify independent prognostic factors. Subgroup analyses were further conducted according to different clinical characteristics to evaluate the differential efficacy of NHT on bPFS, rPFS, and OS across subgroups. Multivariate Cox regression models were employed to calculate interaction P-values (with P-interaction < 0.05 considered statistically significant), and forest plots were used to visualize event rates (%) and HRs for each subgroup. Additionally, multivariate logistic regression was performed to assess the risk of receiving subsequent treatment between the two groups, with adjustment for biopsy Gleason score, pathological T stage, N stage, study center, and year of diagnosis.

All statistical analyses were performed using SPSS version 25.0. Unless otherwise specified, all tests were two-sided, and a P-value < 0.05 was considered statistically significant.

## 3 Results

### 3.1 Baseline patient characteristics

A total of 160 OmPCa patients were enrolled, with 80 patients each in the NHT group and the ST group. All included patients were of Asian (Han Chinese) ethnicity. The median duration of NHT received by patients in the NHT group was 4 (IQR 2-6) months. No statistically significant differences were observed between the two groups regarding baseline characteristics, including age, body mass index (BMI), initial PSA level, initial prostate volume (PV), biopsy Gleason score, clinical T stage, clinical N stage, seminal vesicle invasion status, and number of distant metastases (all P > 0.05; **Table 1**).

**Table 1:** Baseline characteristics of the cohorts.

Characteristics	NHT Group (n=80)	ST Group (n=80)	Statistic	*P* value
Age(years), median (IQR)	67(63,71)	67(64,73)	-1.344	0.179
BMI(kg/m^2^), mean ± SD	25.34 ± 3.11	24.37 ± 3.10	-0.699	0.485
PSA(ng/ml), median (IQR)				
Initial diagnosis	42.67(18.12,117.15)	25.12(15.82,63.44)	-1.832	0.067
Preoperative	0.33(0.08,1.36)	–	–	–
PV(cm^3^), median (IQR)				
Initial diagnosis	45.80(36.63,57.33)	43.44(31.87,51.70)	-1.809	0.070
Preoperative	26.78(20.32,32.48)	–	–	–
Biopsy Gleason score​	9(7,9)	8(7,9)	-1.863	0.063
Biopsy ISUP grade group, n (%)			9.166	0.057
1 (Gleason ≤6)	6(7.5)	0		
2 (Gleason 3 + 4 = 7)	9(11.3)	13(16.3)		
3 (Gleason 4 + 3 = 7)	9(11.3)	9(11.3)		
4 (Gleason 8)	12(15.0)	20(25.0)		
5 (Gleason 9-10)	44(55.0)	38(47.5)		
Clinical T stage, n (%)			0.027	0.870
≤T2c	30(33.3)	29(44.0)		
>T2c	50(66.7)	51(56.0)		
Radiological N stage, n (%)			0.552	0.457
N0	59(74.7)	63(81.0)		
N1	21(25.3)	17(19.0)		
Seminal vesicle invasion, n (%)			2.331	0.127
Yes	30(32.7)	21(38.0)		
No	50(67.3)	59(62.0)		
Number of metastases, median (IQR)	2(1,3)	1(1,2)	-1.465	0.143

SD, standard deviation; IQR, interquartile range; NHT, neoadjuvant hormonal therapy; ST, standard therapy; BMI, body mass index; PSA, prostate-specific antigen; PV, prostate volume.

After NHT treatment, the median PSA level and prostate volume in the NHT group were 0.33 ng/ml (IQR, 0.08–1.36 ng/ml) and 26.78 cm³ (IQR, 20.32–32.48 cm³), respectively, both of which were significantly reduced compared to baseline (both P < 0.05).

### 3.2 Surgical and postoperative pathological information

Compared to the ST group, the NHT group exhibited significantly reduced median operative time (170 min vs. 200 min, P = 0.030) and median blood loss (100 mL vs. 200 mL, P = 0.022). No statistically significant differences were found between the groups in median postoperative hospital stay, median drainage tube removal time, or median catheter removal time (all P > 0.05). Pelvic lymph node dissection was performed in 57 patients in the NHT group and 49 patients in the ST group, with no significant difference between the groups (P = 0.181). All surgeries were successfully completed without the need for blood transfusion or conversion to open laparotomy. Within 30 days postoperatively, 5 patients in the NHT group experienced surgery-related complications (Clavien-Dindo grade II: 4 cases; grade III: 1 case), while 6 patients in the ST group experienced complications (Clavien-Dindo grade I: 1 case; grade II: 4 cases; grade III: 1 case). No severe complications or deaths occurred in either group.

Postoperative pathological examination showed a significantly lower PSM rate in the NHT group compared to the ST group (17.5% vs. 37.5%, P = 0.005). In the NHT group, pCR and MRD were achieved in 1 (1.3%) and 3 (3.8%) patients, respectively, whereas no patients in the ST group attained pCR or MRD. Pathological downstaging of T stage was observed in 39 (48.8%) patients in the NHT group, respectively, which was significantly higher than the 5 (6.3%, P < 0.001) patients in the ST group. Among patients who underwent lymph node dissection, postoperative pathology showed pN1 disease in 9 patients (15.8%) in the NHT group and 11 patients (22.4%) in the ST group, with no statistically significant difference between the two groups (P = 0.382). As shown in **Table 2**.

**Table 2:** Surgical and postoperative pathological information of the cohorts.

Characteristics	NHT Group (n=80)	ST Group (n=80)	Statistic	*P* value
Operative time (min), median (IQR)	170(120,220)	200(150,250)	-2.164	0.030
Blood loss (mL), median (IQR)	100(50,200)	200(100,300)	-2.297	0.022
Transfusion volume (mL), median (IQR)	0	0	–	–
Postoperative hospital stay (d), median (IQR)	7(4,8)	7(5,10)	-1.657	0.098
Drain removal time (d), median (IQR)	4(3,6)	5(3,8)	-1.933	0.053
Catheter removal time (d), median (IQR)	14(14,14)	14(14,14)	-0.885	0.376
Lymph node dissection, n (%)			1.789	0.181
Yes	57(71.3)	49(61.3)		
No	23(28.8)	31(38.8)		
Clavien-Dindo classification, n (%)			1.667	0.435
Grade I	0	1(16.7)		
Grade II	4(80.0)	4(66.7)		
Grade III	1(20.0)	1(16.7)		
Grade IV/V	0	0		
pN1, n (%)	9(15.8)	11(22.4)	0.763	0.382
Positive surgical margin, n (%)	14(17.5)	30(37.5)	8.025	0.005
Pathological complete response, n (%)	1(1.3)	0	1.006	0.316
Minimal residual disease, n (%)	3(3.8)	0	3.057	0.080
Pathological T stage change, n (%)			43.185	<0.001
Downstaging	39(48.8)	5(6.3)		
No change	38(47.5)	53(66.3)		
Upstaging	3(3.8)	22(27.5)		

SD, standard deviation; IQR, interquartile range; NHT, neoadjuvant hormonal therapy; ST, standard therapy.

### 3.3 Oncological outcome

All patients underwent postoperative follow-up for oncological outcome assessment. The median postoperative follow-up time was 49 (IQR 35-73) months for the NHT group and 54 (IQR 46-73) months for the ST group, showing no significant difference (P = 0.127). During follow-up, significantly more patients in the NHT group achieved a radical-level PSA compared to the ST group (83.8% vs. 65.0%, P = 0.007). Furthermore, the median time to nadir PSA was significantly shorter in the NHT group than in the ST group (2 months vs. 3 months, P = 0.002).

**Figure 1:**
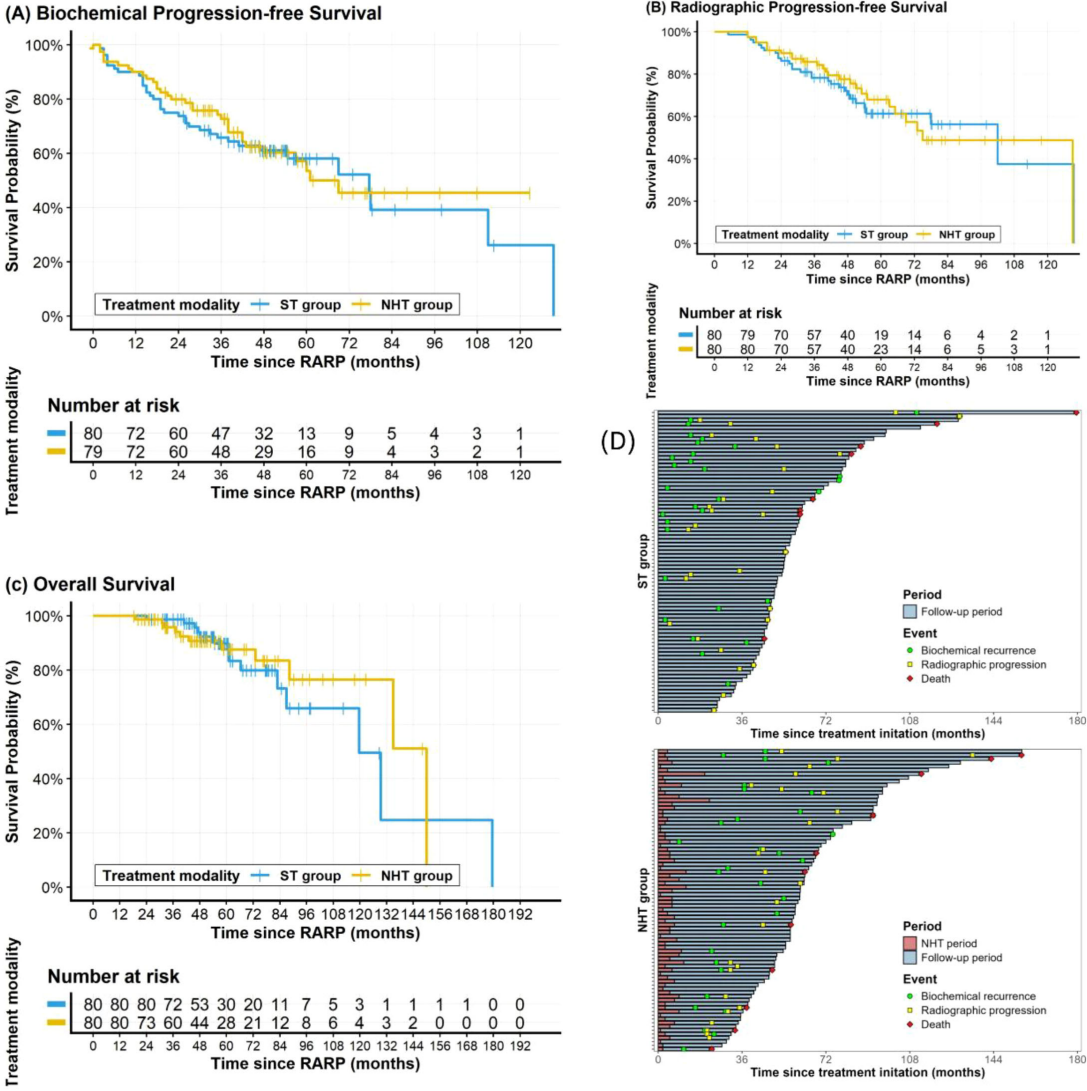
Kaplan-Meier curves of oncological outcomes: **(A)** biochemical progression-free survival, **(B)** radiographic progression-free survival, **(C)** overall survival, **(D)** swimmer’s plot of the cohorts. NHT, neoadjuvant hormonal therapy; ST, standard therapy.

The Kaplan-Meier survival curves for bPFS, rPFS, and OS are shown in **Figure 1**. By the last follow-up, BCR had occurred in 32 (40.0%) patients in the NHT group and 36 (45.0%) in the ST group; the 3-year bPFS rates were 68.6% and 64.3%, respectively. Radiological progression occurred in 26 (32.5%) patients in the NHT group and 29 (36.3%) in the ST group during follow-up, with 3-year rPFS rates of 77.7% and 76.8%, respectively. Death occurred in 11 (13.8%) patients in the NHT group and 14 (17.5%) in the ST group; the 3-year OS rates were 95.8% and 98.8%, respectively. Overall, no statistically significant differences were observed between the two groups in bPFS, rPFS, or OS (all P > 0.05). Univariate and multivariate Cox proportional hazards models showed that NHT was not a protective factor for bPFS, rPFS, or OS (**Supplementary Tables 2**-**4**).

This study used bPFS, rPFS, and OS as primary outcome measures. Subgroup analyses were performed for the variables listed in **Supplementary Tables 1**-**3** to assess the interaction between each subgroup and NHT. Subgroup analysis revealed that biopsy Gleason score, clinical T stage, and seminal vesicle invasion all had significant interactions with NHT regarding bPFS, rPFS, and OS (all P for interaction < 0.05). Specifically, in subgroups with a biopsy Gleason score > 7, clinical T stage > T2c, and the presence of seminal vesicle invasion, NHT significantly prolonged bPFS (Gleason score ≥ 8: HR = 0.42, 95% CI: 0.22–0.78, P = 0.006; clinical T stage > T2c: HR = 0.42, 95% CI: 0.21–0.85, P = 0.016; seminal vesicle invasion: HR = 0.33, 95% CI: 0.13–0.87, P = 0.026) (**Supplementary Figure 1**), rPFS (Gleason score ≥8: HR = 0.37, 95% CI: 0.18–0.73, P = 0.004; clinical T stage > T2c: HR = 0.44, 95% CI: 0.21–0.94, P = 0.034; seminal vesicle invasion: HR = 0.35, 95% CI: 0.13–0.92, P = 0.033) (**Supplementary Figure 2**), and OS (Gleason score ≥8: HR = 0.30, 95% CI: 0.11–0.84, P = 0.021; clinical T stage > T2c: HR = 0.42, 95% CI: 0.19–0.95, P = 0.037; seminal vesicle invasion: HR = 0.27, 95% CI: 0.08–0.90, P = 0.033) (**Supplementary Figure 3**) in patients with OmPCa. The interactions between all other variables and NHT treatment were not statistically significant (all P-interaction > 0.05).

### 3.4 Subsequent treatment

During follow-up, 67.5% (54/80) and 52.5% (42/80) of patients in the NHT and ST groups, respectively, did not receive any additional treatment (P = 0.031). In the NHT and ST groups, 13.8% (11/80) and 28.8% (23/80) of patients received novel endocrine therapy agents such as abiraterone (P = 0.020), 7.5% (6/80) and 6.3% (5/80) underwent chemotherapy (P = 0.755), 5.0% (4/80) and 12.5% (10/80) received adjuvant radiotherapy, and 11.3% (9/80) and 21.3% (17/80) received salvage radiotherapy. The differences in these subsequent treatment modalities between the groups were statistically significant (P = 0.020; **Supplementary Table 5**).

After adjusting for potential confounders including biopsy Gleason score, pathological stage, study center, and year of diagnosis using multivariable logistic regression, NHT remained independently associated with a lower risk of subsequent treatment (OR = 0.40, 95% CI 0.19–0.85, P = 0.017).

### 3.5 ADT-related adverse events

**Table 3** presents the adverse events associated with ADT that occurred during the postoperative follow-up period. The assessment of ADT-related adverse events was limited to patients who had no corresponding symptoms at baseline. Specifically, in the NHT group and the ST group, 11 and 13 patients, respectively, had pre-existing sexual dysfunction before treatment, while 20 and 17 patients had pre-existing hypertension; these patients were excluded from the statistical analysis of the corresponding adverse events. The results showed that common adverse events in both groups included sexual dysfunction, hot flashes, and fatigue, and none of the ADT-related adverse events demonstrated statistically significant differences between the two groups (all P > 0.05). No severe ADT-related adverse event occurred in either group.

**Table 3:** Adverse events associated with ADT of the cohorts.

Event	NHT Group	ST Group	Statistic	*P* value
Hot flush, n (%)	33(41.3)	31(38.8)	0.104	0.747
Fatigue, n (%)	29(36.3)	28(35.0)	0.027	0.869
Gynecomastia, n (%)	13(16.3)	17(21.3)	0.656	0.418
Anemia, n (%)	11(13.8)	9(11.3)	0.229	0.633
Sexual dysfunction, n (%)	58(84.1)	55(82.1)	0.094	0.759
Weight increased, n (%)	12(15.0)	8(10.0)	0.914	0.339
Back pain, n (%)	10(12.5)	9(11.3)	0.060	0.807
Pain in an arm or leg, n (%)	10(12.5)	8(10.0)	0.250	0.617
Bone pain, n (%)	4(5.0)	5(6.25)	0.118	0.732
Fracture, n (%)	1(1.3)	0(0.0)	1.006	0.316
Urinary retention, n (%)	1(1.3)	2(2.5)	0.340	0.560
Hypertension, n (%)	11(18.3)	10(15.9)	0.131	0.717
Other cardiovascular events, n (%)	0(0.0)	1(1.3)	1.006	0.316

NHT, neoadjuvant hormonal therapy; ST, standard therapy.

## 4 Discussion

OmPCa represents a distinct subset of PCa characterized by a limited metastatic burden. Although there is no universally established optimal treatment strategy for OmPCa, a growing consensus supports the use of aggressive therapeutic interventions to delay its progression to polymetastatic disease and alter its natural history (18, 19). Prostate cancer is a hormone-dependent malignancy (20); testosterone and its derivatives bind to the androgen receptor in both prostatic epithelial and stromal cells, activating multiple intracellular signaling pathways that promote tumor cell proliferation and disease progression (21). This study aims to evaluate the efficacy of NHT in OmPCa patients undergoing RARP combined with postoperative endocrine therapy, thereby providing evidence to support clinical decision-making regarding the timing of surgical intervention for OmPCa.

The role of RP in the management of OmPCa has garnered increasing attention in recent years. Several retrospective studies and meta-analyses have suggested that local treatment of the primary tumor, including RP, may confer survival benefits in appropriately selected patients with OmPCa (10, 19, 22). The rationale for RP includes the reduction of primary tumor burden, potential elimination of sources for further metastatic seeding, and enhanced responsiveness to systemic therapy. In our study, all patients underwent RARP, which aligns with the cytoreductive principle. However, the key distinction lies in the use of NHT prior to surgery in the experimental group.

Although this study did not observe a long-term survival benefit from NHT in the overall OmPCa population, subgroup analysis suggested a potential survival advantage in patients with high-risk features, such as biopsy Gleason score ≥8, clinical stage ≥T3, and seminal vesicle invasion. We identified a significant interaction between these subgroups and treatment modality, wherein NHT markedly reduced the risk of recurrence in patients with high tumor burden and aggressive disease, and demonstrated a trend toward improved OS.

This may be attributed to the highly aggressive nature of tumors in these patients, which are more prone to local invasion, thereby increasing the difficulty of achieving negative surgical margins during resection (23, 24). For this subset of OmPCa patients, NHT may maximize reduction of primary tumor volume and downstage the disease, potentially rendering previously unresectable or high-risk tumors more amenable to complete excision and increasing the rate of negative margins. Concurrently, this subgroup of OmPCa harbors a higher metastatic potential. NHT may induce rapid and potent systemic suppression by inhibiting the androgen signaling pathway, thereby delaying the activation of micrometastases. Thereby, more effective control of systemic dissemination may be achieved prior to systemic therapy, ultimately translating into a survival benefit.

However, these findings must be interpreted with caution. Given that bPFS, rPFS, and OS did not reach statistical significance in the overall population of this study, and that subgroup analyses were not adjusted for multiple comparisons, the above observations are inherently hypothesis-generating. Future studies with rigorous designs, incorporating variables such as Gleason score and T stage as stratification factors, are warranted to validate the precise efficacy of NHT in specific patient subgroups.

In this study, NHT significantly reduced the incidence of PSM and induced pathological T-stage downstaging in patients with OmPCa. These findings align with previously reported research (14, 25). Compared to the ST group, the NHT group demonstrated significant advantages in increasing the proportion of patients achieving a radical-level PSA (83.8% vs. 65.0%, P = 0.007), and shortening the median time to nadir PSA (2 months vs. 3 months, P = 0.002). Previous research has confirmed that pathological downstaging is an independent favorable prognostic factor in PCa (26). Furthermore, the PSA nadir and the time to reach it are also significant predictors of survival outcomes in metastatic prostate cancer patients (27–30). Therefore, these results further suggest that NHT may confer potential survival benefits for OmPCa patients.

Previous studies have reported that NHT may increase the risk of prostate inflammatory infiltration, stromal fibrosis, and seminal vesicle adhesions, potentially increasing surgical difficulty (31, 32). However, this study found that the median operative time and median blood loss were significantly lower in the NHT group compared to the ST group, with no significant differences in postoperative recovery indicators or short-term complication rates. Consequently, we conclude that NHT did not increase the difficulty of RARP. It may even improve certain perioperative metrics, possibly by reducing tumor angiogenesis and creating clearer tissue planes, thereby facilitating dissection. These findings align with the views of scholars such as Labrie (33) and Pu et al. (34).

Furthermore, we observed with interest that the additional treatment rate in the NHT group was significantly lower than that in the ST group (32.5% vs. 47.5%, P = 0.031; **Supplementary Table 4**). This finding suggests that although NHT did not significantly prolong overall survival within the current follow-up period, it may have altered the disease trajectory by providing more durable local and systemic control. We hypothesize that the lower positive surgical margin rate and higher pathological downstaging observed in the NHT group translated into a reduced residual tumor burden, thereby delaying or even obviating the need for subsequent salvage therapies.

The clinical implications of this reduction are substantial. Previous literature has reported that leukopenia, as the most common adverse reaction to chemotherapy, can occur in up to 41.7% of patients and increases the risk of infection (35). Meanwhile, radiotherapy is often associated with genitourinary complications such as urinary incontinence, dysuria, urethral obstruction, and recurrent urinary bleeding, which significantly impair long-term quality of life (36). Therefore, NHT may hold important clinical value in reducing the need for additional treatments, thereby helping to mitigate related complications and alleviate the associated financial burden on patients.

From a patient-centered perspective, the value of NHT should therefore be evaluated not solely by survival extension, but also by its ability to achieve “treatment de-intensification”—that is, maintaining disease control with fewer subsequent interventions. This is particularly relevant in the oligometastatic setting, where the goal of treatment increasingly balances oncologic control with preservation of quality of life. While longer follow-up is warranted to determine whether this reduction in salvage therapy ultimately translates into a survival benefit, the current findings support NHT as a strategy that may improve the overall treatment journey for patients with OmPCa undergoing RARP.

Regarding safety, ADT is associated with well-established long-term risks, including cardiovascular disease, osteoporosis, and metabolic syndrome (37, 38). Furthermore, studies have elucidated that even short-term NHT followed by radical prostatectomy and pelvic lymph node dissection may confer an increased risk of thromboembolic events (39). However, in the present cohort, no thromboembolic events were observed in patients who received NHT compared with those who underwent standard treatment alone, and no statistically significant increase in other ADT-related adverse events was noted. This finding may be attributed to the relatively short duration of the NHT regimen, or it may reflect insufficient statistical power to detect such events in this study. Given that ADT-related adverse events typically exhibit a clear dose–time dependency (40), a brief additional exposure to NHT is unlikely to yield a detectable incremental risk against the backdrop of lifelong ADT—a cumulative pathophysiological process common to both groups.

This study has the following limitations: Firstly, as a multicenter retrospective study, the treatment regimens for ADT and subsequent salvage therapeutic strategies were not fully standardized. Furthermore, the assessment of ADT-related adverse events was susceptible to recall bias and discrepancies in documentation, leading to inherent limitations in accuracy and consistency. Secondly, the follow-up duration was insufficiently long, as the median bPFS, median rPFS, and median OS had not been reached in either group, potentially affecting the precise assessment of long-term survival benefits. Future studies with longer follow-up are needed for validation. Additionally, the study spanned a considerable timeframe, during which advancements in PET/CT tracer technology and the evolution of AR inhibitors may have influenced the outcomes. Moreover, this study exclusively enrolled patients with bone metastases and of Asian ethnicity, excluding cases with lymph node involvement or from other racial backgrounds, thereby limiting the generalizability of the results to broader oligometastatic populations. Finally, because prostate specimens after NHT are often unsuitable for accurate Gleason score assessment, this study could not analyze the impact of NHT on Gleason score.

## 5 Conclusion

In conclusion, this study confirms that for OmPCa patients, NHT significantly improves certain perioperative outcomes and reduces the need for subsequent salvage therapies. It demonstrates potential oncological benefits, particularly in those with a biopsy Gleason score ≥8 or clinical T-stage≥T3. Based on these findings, NHT represents a safe and feasible treatment strategy prior to RARP for OmPCa patients. However, its definitive long-term oncological benefits require further validation through studies with longer follow-up durations.

## Data Availability

The original contributions presented in the study are included in the article/**Supplementary Material**. Further inquiries can be directed to the corresponding authors.

## Supplementary material

Supplementary Table 1 PET tracer types used across participating centers and study periods

Supplementary Table 2 Univariate and multivariate analyses of factors associated with biochemical progression-free survival. HR, hazard ratios; CI, confidence intervals; BMI, body mass index; PSA, prostate-specific antigen; PV, prostate volume; NHT, neoadjuvant hormonal therapy.

Supplementary Table 3 Univariate and multivariate analyses of factors associated with radiographic progression-free survival. HR, hazard ratios; CI, confidence intervals; BMI, body mass index; PSA, prostate-specific antigen; PV, prostate volume; NHT, neoadjuvant hormonal therapy.

Supplementary Table 4 Univariate and multivariate analyses of factors associated with overall survival. HR, hazard ratios; CI, confidence intervals; BMI, body mass index; PSA, prostate-specific antigen; PV, prostate volume; NHT, neoadjuvant hormonal therapy.

Supplementary Table 5 Subsequent treatments of the cohorts. SD, standard deviation; IQR, interquartile range; NHT, neoadjuvant hormonal therapy; ST, standard therapy.

Supplementary Figure 1 Forest plot for the interaction analysis of the effect of neoadjuvant hormonal therapy on biochemical progression-free survival across subgroups. BMI, body mass index; PSA, prostate-specific antigen; PV, prostate volume.

Supplementary Figure 2 Forest plot for the interaction analysis of the effect of neoadjuvant hormonal therapy on radiographic progression-free survival across subgroups. BMI, body mass index; PSA, prostate-specific antigen; PV, prostate volume.

Supplementary Figure 3 Forest plot for the interaction analysis of the effect of neoadjuvant hormonal therapy on overall survival across subgroups. BMI, body mass index, PSA, prostate-specific antigen; PV, prostate volume.
