# Supplementary material for: Efficacy of neoadjuvant hormonal therapy combined with robot-assisted radical prostatectomy for oligometastatic prostate cancer: a multicenter retrospective study
Source: Front Oncol. 2026 Mar 26;16:1765517. doi: 10.3389/fonc.2026.1765517 (PMC13062178; doi:10.3389/fonc.2026.1765517)
Supplement: Supplementary Table 1 — PET tracer types used across participating centers and study periods [file Table1.docx]

Supplementary Table 1: PET tracer types used across participating centers and study periods

| Center | Period | PET tracer type |
| --- | --- | --- |
| The First, Third, Sixth Medical Centers of Chinese PLA General Hospital | 2010-2014 | ^18^F-FDG |
|  | 2015-2016 | ^11^C-Choline |
|  | 2017-2020 | ^68^Ga-PSMA |
|  | 2021-2023 | ^18^F-PSMA |
| The First Affiliated Hospital of Nanchang University | 2010-2016 | ^18^F-FDG |
|  | 2017-2021 | ^68^Ga-PSMA |
|  | 2022-2023 | ^18^F-PSMA |
| The First Affiliated Hospital of Soochow University | 2010-2014 | ^18^F-FDG |
|  | 2015-2016 | ^11^C-Choline |
|  | 2017-2021 | ^68^Ga-PSMA |
|  | 2022-2023 | ^18^F-PSMA |
