# Supplementary material for: Efficacy of neoadjuvant hormonal therapy combined with robot-assisted radical prostatectomy for oligometastatic prostate cancer: a multicenter retrospective study
Source: Front Oncol. 2026 Mar 26;16:1765517. doi: 10.3389/fonc.2026.1765517 (PMC13062178; doi:10.3389/fonc.2026.1765517)
Supplement: Supplementary Table 2 — Univariate and multivariate analyses of factors associated with biochemical progression-free survival. HR, hazard ratios; CI, confidence intervals; BMI, body mass index; PSA, prostate-specific antigen; PV, prostate volume; NHT, neoadjuvant hormonal therapy. [file Table2.docx]

Supplementary Table 2: Univariate and multivariate analyses of factors associated with biochemical progression-free survival

| Variable | Univariable | | Multivariable | |
| --- | --- | --- | --- | --- |
|  | HR (95% CI) | *p* value | HR (95% CI) | *p* value |
| Age | 1.01 (0.97, 1.04) | 0.735 | - | - |
| BMI | 0.99 (0.91, 1.07) | 0.715 | - | - |
| Initial PSA | 1.00 (0.99, 1.01) | 0.105 | - | - |
| Initial PV | 1.01 (0.99, 1.03) | 0.353 | - | - |
| Biopsy Gleason score | 1.68 (1.26, 2.24) | <0.001 | 1.71 (1.25, 2.35) | 0.001 |
| Clinical T stage (>T2c vs ≤T2c) | 0.86 (0.52, 1.40) | 0.532 | - | - |
| Radiological N stage (N1 vs N0) | 1.79 (1.06, 3.01) | 0.029 | 1.46 (0.86, 2.50) | 0.164 |
| Seminal vesicle invasion (Yes vs No) | 1.16 (0.68, 1.98) | 0.587 | - | - |
| Number of metastases | 1.11 (0.92, 1.34) | 0.290 | - | - |
| Treatment (NHT VS non-NHT) | 0.89 (0.55, 1.44) | 0.629 | 0.70 (0.42, 1.17) | 0.169 |

Abbreviations: HR = hazard ratios, CI = confidence intervals, BMI = body mass index, PSA = prostate-specific antigen, PV = prostate volume, NHT = neoadjuvant hormonal therapy.
