# Supplementary material for: Efficacy of neoadjuvant hormonal therapy combined with robot-assisted radical prostatectomy for oligometastatic prostate cancer: a multicenter retrospective study
Source: Front Oncol. 2026 Mar 26;16:1765517. doi: 10.3389/fonc.2026.1765517 (PMC13062178; doi:10.3389/fonc.2026.1765517)
Supplement: Supplementary Table 3 — Univariate and multivariate analyses of factors associated with radiographic progression-free survival. HR, hazard ratios; CI, confidence intervals; BMI, body mass index; PSA, prostate-specific antigen; PV, prostate volume; NHT, neoadjuvant hormonal therapy. [file Table3.docx]

Supplementary Table 3: Univariate and multivariate analyses of factors associated with radiographic progression-free survival

| Variable | Univariable | | Multivariable | |
| --- | --- | --- | --- | --- |
|  | HR (95% CI) | *p* value | HR (95% CI) | *p* value |
| Age | 1.00 (0.96, 1.03) | 0.774 | - | - |
| BMI | 1,03 (0.94, 1.13) | 0.491 | - | - |
| Initial PSA | 1.00 (0.99, 1.01) | 0.738 | - | - |
| Initial PV | 1.00 (0.98, 1.02) | 0.667 | - | - |
| Biopsy Gleason score | 1.74 (1.25, 2.43) | 0.001 | 1.85 (1.29, 2.66) | 0.001 |
| Clinical T stage (>T2c vs ≤T2c) | 0.83 (0.48, 1.44) | 0.506 | - | - |
| Radiological N stage (N1 vs N0) | 1.35 (0.74, 2.46) | 0.325 | - | - |
| Seminal vesicle invasion (Yes vs No) | 1.48 (0.84, 2.62) | 0.175 | - | - |
| Number of metastases | 1.01 (0.82, 1.24) | 0.950 | - | - |
| Treatment (NHT VS non-NHT) | 0.92 (0.54, 1.57) | 0.767 | 0.72 (0.41, 1.27) | 0.257 |

Abbreviations: HR = hazard ratios, CI = confidence intervals, BMI = body mass index, PSA = prostate-specific antigen, PV = prostate volume, NHT = neoadjuvant hormonal therapy.
