# Supplementary material for: Efficacy of neoadjuvant hormonal therapy combined with robot-assisted radical prostatectomy for oligometastatic prostate cancer: a multicenter retrospective study
Source: Front Oncol. 2026 Mar 26;16:1765517. doi: 10.3389/fonc.2026.1765517 (PMC13062178; doi:10.3389/fonc.2026.1765517)
Supplement: Supplementary Table 4 — Univariate and multivariate analyses of factors associated with overall survival. HR, hazard ratios; CI, confidence intervals; BMI, body mass index; PSA, prostate-specific antigen; PV, prostate volume; NHT, neoadjuvant hormonal therapy. [file Table4.docx]

Supplementary Table 4: Univariate and multivariate analyses of factors associated with overall survival

| Variable | Univariable | | Multivariable | |
| --- | --- | --- | --- | --- |
|  | HR (95% CI) | *p* value | HR (95% CI) | *p* value |
| Age | 1.08 (1.01, 1.15) | 0.020 | 1.07 (1.00, 1.15) | 0.059 |
| BMI | 0.96 (0.83, 1.11) | 0.588 | - | - |
| Initial PSA | 1.00 (0.99, 1.01) | 0.820 | - | - |
| Initial PV | 1.01 (0.98, 1.04) | 0.576 | - | - |
| Biopsy Gleason score | 2.78 (1.51, 5.13) | 0.001 | 3.02 (1.49, 6.12) | 0.002 |
| Clinical T stage (>T2c vs ≤T2c) | 0.80 (0.36, 1.81) | 0.597 | - | - |
| Radiological N stage (N1 vs N0) | 2.35 (0.96, 5.78) | 0.063 | 1.43 (0.57, 3.61) | 0.451 |
| Seminal vesicle invasion (Yes vs No) | 0.87 (0.34, 2.23) | 0.777 | - | - |
| Number of metastases | 1.32 (0.98, 1.78) | 0.070 | 1.31 (0.97, 1.77) | 0.083 |
| Treatment (NHT VS non-NHT) | 0.85 (0.38, 1.91) | 0.694 | 0.73 (0.29, 1.83) | 0.505 |

Abbreviations: HR = hazard ratios, CI = confidence intervals, BMI = body mass index, PSA = prostate-specific antigen, PV = prostate volume, NHT = neoadjuvant hormonal therapy.
