# Supplementary material for: Efficacy of neoadjuvant hormonal therapy combined with robot-assisted radical prostatectomy for oligometastatic prostate cancer: a multicenter retrospective study
Source: Front Oncol. 2026 Mar 26;16:1765517. doi: 10.3389/fonc.2026.1765517 (PMC13062178; doi:10.3389/fonc.2026.1765517)
Supplement: Supplementary Table 5 — Subsequent treatments of the cohorts. SD, standard deviation; IQR, interquartile range; NHT, neoadjuvant hormonal therapy; ST, standard therapy. [file Table5.docx]

Supplementary Table 5: Subsequent treatments of the cohorts

| Characteristics | NHT Group (n=80) | ST Group (n=80) | Statistic | *p* value |
| --- | --- | --- | --- | --- |
| No further treatment, n(%) | 54 (67.5) | 42 (52.5) | 4.637 | 0.031 |
| Novel hormonal therapy, n(%) | 11 (13.8) | 23 (28.8) | 5.378 | 0.020 |
| Chemotherapy, n(%) | 6 (7.5) | 5 (6.3) | 0.098 | 0.755 |
| Radiotherapy, n(%) |  |  | 6.614 | 0.037 |
| Adjuvant radiotherapy | 4 (5.0) | 10 (12.5) |  |  |
| Salvage radiotherapy | 9 (11.3) | 17 (21.3) |  |  |
| No radiotherapy | 69 (86.3) | 55 (68.8) |  |  |
| Overall |  |  | 9.843 | 0.020 |

Abbreviations: SD = standard deviation, IQR = interquartile range, NHT = neoadjuvant hormonal therapy, ST = standard therapy.
