# Supplementary material for: Efficacy of neoadjuvant hormonal therapy combined with robot-assisted radical prostatectomy for oligometastatic prostate cancer: a multicenter retrospective study
Source: Front Oncol. 2026 Mar 26;16:1765517. doi: 10.3389/fonc.2026.1765517 (PMC13062178; doi:10.3389/fonc.2026.1765517)
Supplement: Supplementary Figure 1 — Forest plot for the interaction analysis of the effect of neoadjuvant hormonal therapy on biochemical progression-free survival across subgroups. BMI, body mass index; PSA, prostate-specific antigen; PV, prostate volume. [file Image1.pdf]

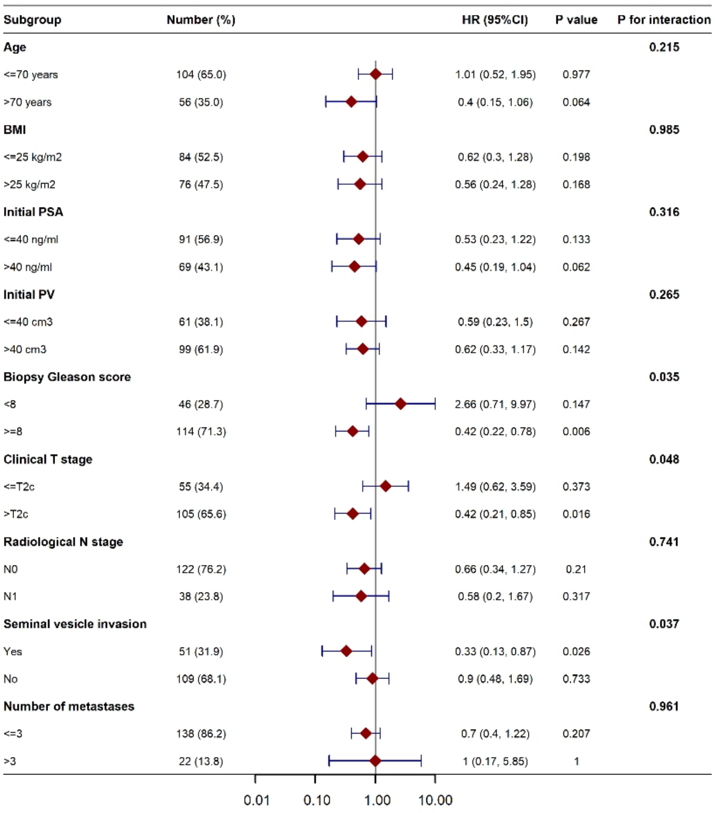

Supplementary Figure 1: Forest plot for the interaction analysis of the effect of neoadjuvant hormonal therapy on biochemical progression-free survival across subgroups.

Abbreviations: BMI = body mass index, PSA = prostate-specific antigen, PV = prostate volume.
